# Evaluation of the knowledge and correct use of metered-dose inhalers by healthcare professionals and medical students in Gauteng Province

**DOI:** 10.7196/AJTCCM.2019.v25i3.003

**Published:** 2019-09-17

**Authors:** H M Maepa, M L Wong, C N Menezes

**Affiliations:** 1 Division of Pulmonology, Department of Internal Medicine, Chris Hani Baragwanath Academic Hospital, Johannesburg, South Africa; 2 Division of Infectious Diseases, Department of Internal Medicine, Chris Hani Baragwanath Academic Hospital, Johannesburg, South Africa; 3 Faculty of Health Sciences, University of the Witwatersrand, Johannesburg, South Africa

**Keywords:** MDI, inhaler technique, health care professionals, poor inhaler technique

## Abstract

**Background:**

The Global Initiative for Asthma (GINA) estimates that South Africa (SA) has over 3.9 million asthma sufferers, of whom 1.5% die of the condition annually. SA has the world’s fourth highest asthma death rate among 5 - 35-year-olds. Chronic obstructive pulmonary disease (COPD) will be the third leading cause of death globally by 2030, and will surpass HIV/AIDS in Africa. Uncontrolled asthma and COPD are frequent causes of emergency department visits and hospital admissions. Poor metered-dose inhaler (MDI) technique is probably a major contributory cause. It is the responsibility of all treating doctors and healthcare professionals to educate patients on inhaled therapy with the correct MDI technique, as well as to routinely check and repeatedly demonstrate the technique to them.

**Objectives:**

This study evaluated study participants’ knowledge of MDI technique, and their compliance in checking and demonstrating MDI use to patients prescribed inhaled therapy. The study participants included doctors, nurses and final-year medical students at Helen Joseph Hospital and Chris Hani Baragwanath Academic Hospital, in the Departments of Internal Medicine and Emergency Medicine, and the Division of Pulmonology.

**Methods:**

A total of 195 study participants volunteered to take part in the study. A questionnaire was administered to participants to gauge their perceptions, level of knowledge and understanding of MDI technique. They were then requested to demonstrate correct inhaler technique via a placebo MDI. Participants’ use of a placebo MDI was evaluated by a scoring system.

**Results:**

The total sample of 195 comprised 130 (67%) female and 65 (33%) male participants. Of these, 133 (68%) were qualified medical staff, and 62 were final-year medical students. Only 32 (16%) could demonstrate correct MDI technique. Over 50% of participants did not demonstrate MDI technique to patients, or check their patients’ technique at every hospital-related visit.

**Conclusion:**

Healthcare professionals and final-year medical students have poor knowledge of inhaler technique and are ill-prepared to teach patients. Also of concern is that the majority do not routinely demonstrate or observe patients’ inhaler technique.

## Background

The prevalence of chronic lung disease is increasing in all parts of the globe. Three hundred million people have asthma and 210 million people have chronic obstructive pulmonary disease (COPD) worldwide.^[1,2]^ The Global Initiative for Asthma (GINA) estimates that South Africa (SA) has over 3.9 million asthma sufferers, of whom 1.5% die of the condition annually. SA has the world’s fourth highest asthma death rate among 5 - 35-year-olds.^[2–4]^ The latest World Health Organization (WHO) statistics in 2005 confirmed that ~5% of all deaths globally are due to COPD.^[4,5]^ Ninety percent of these deaths are thought to occur in low- and middle-income countries such as SA. COPD will be the third leading cause of death globally by 2030, and will surpass HIV/AIDS in Africa.^[4,5]^

Inhaled therapy for chronic airway diseases (bronchodilators and corticosteroids) are most commonly administered by metered-dose inhalers (MDIs). They are crucial to the management of both asthma and COPD. MDIs deliver a specific amount of medication, which is stored in a highly pressurised canister, to the lungs, in the form of a short burst of aerosolised drug, self-administered by the patient via inhalation. When used correctly with adequate dosing, they have been shown to provide symptomatic relief, improve lung function and reduce exacerbations.^[2–4]^ MDIs, however, are technically more difficult to use than dry-powder inhalers, as they require good simultaneous coordination between the handling of the device, and respiration. Patients are therefore more prone to make a critical error, as compared with patients using less technically difficult inhaler devices.^[6]^

Research reveals that many patients do not receive regular counselling or physical demonstration of correct inhaler technique.^[6]^ Physicians have been shown to have poor compliance with checking and demonstrating MDI use to patients who are prescribed inhaled therapy.^[7]^

The present study was conducted to evaluate the knowledge of MDI technique by healthcare professionals (HCPs) and final-year medical students, as well as their compliance in checking and demonstrating MDI technique to patients on inhaled therapy. The burden of asthma and COPD is on the rise in SA, and greater efforts should be made to ensure that patients are taught correct use of MDIs, and that full compliance with treatment guidelines by HCPs is practised.

## Materials and methods

### Study population and data collection

This was a prospective study of HCPs comprising medical doctors and nurses in varying ranks, as well as final-year medical students working in the Departments of Internal Medicine and Emergency Medicine, and the Division of Pulmonology, at Helen Joseph Hospital (HJH) and Chris Hani Baragwanath Academic Hospital (CHBAH).

A total of 195 study participants volunteered to take part in the study. A questionnaire was administered to the study participants to gauge their perceptions, level of knowledge and understanding of MDI technique. They were then requested to demonstrate correct inhaler technique via a placebo MDI. Correct inhaler technique involves 6 manoeuvres in the following sequence: (i) shaking the canister; (ii) exhaling to residual volume; (iii) coordinated simultaneous activation of the MDI with (iv) initiation of inspiration; (v) inhaling the aerosol through the mouth, slowly and deeply over 5 to 6 seconds; followed by (vi) holding the breath for at least 5 seconds prior to exhalation.

Each of the 6 manoeuvres required for correct MDI technique demonstrated by the participant was allocated 1 point. As each manoeuvre is important in adequate delivery of inhaled medication, any component incorrectly performed was regarded as a critical error. The total for each participant was calculated. All errors were documented.

### Statistical analysis

All collected data were entered into an Excel database. The categorical data were analysed using STATA 10.1 statistics software package (StataCorp, USA). Fisher’s exact test was used to detect statistically significant associations between knowledge of MDI use by the different study participants and their compliance in checking and demonstrating MDI use to patients.

## Results

### Demographics

Most of the study participants interviewed were female (n=130) (Table 1). There was no statistical difference in questionnaire responses or demonstration of MDI technique between male and female participants (p=0.2298) (Table 1).

A total of 62 final-year medical students studying at the University of the Witwatersrand and 133 qualified HCPs were interviewed. A third of the HCPs performed their undergraduate studies at the University of the Witwatersrand, 55% studied at other SA universities, and 12% studied outside SA. Only 15 (11%) of the total HCP pool had been qualified for more than 10 years (Table 1). A further 51 (39.2%) of the study participants had been qualified for between 5 and 10 years, and 48.6% for less than 5 years. Irrespective of their years of experience, rank or place of undergraduate studies, there was no statistical difference in adequate knowledge of MDI use, as well as compliance in checking and demonstrating MDI technique to patients. Only eight (4%) study participants were themselves asthmatics on inhaled therapy. There was no statistical significance in their knowledge of inhaler technique when compared with the rest of the study participants.

### HCPs and final-year medical students’ performance of MDI technique

**Fig. 1:**
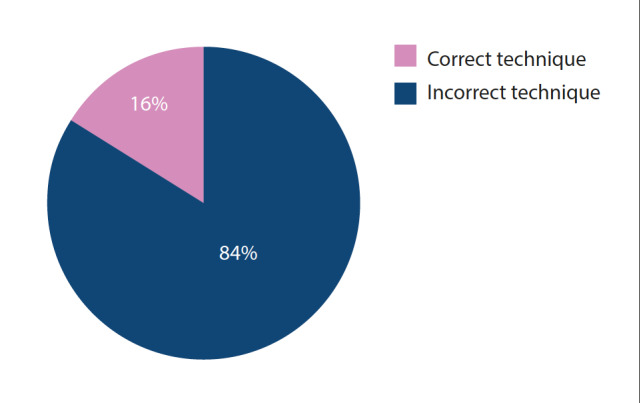
Study participants’ metered-dose inhaler technique (MDI) via a placebo MDI. (Correct MDI technique refers to all 6 manoeuvres adequately performed for effective drug delivery.)

Only 16% of all study participants were able to perform the technique correctly (Fig. 1). There was no difference in scores between medical students and HCPs when assessing whether they had adequate inhaler technique or not (p=0.5243).

### Factors predisposing to poor knowledge of MDI technique

All HCPs and final-year medical students who were interviewed acknowledged the importance of having good knowledge of MDI technique. They also showed good insight into how correct MDI use significantly influences disease control and the reduction of morbidity and mortality. The study showed that there was no statistically significant difference between final-year medical students and qualified HCPs (including nurses) on their knowledge of correct MDI use.

**Fig. 2:**
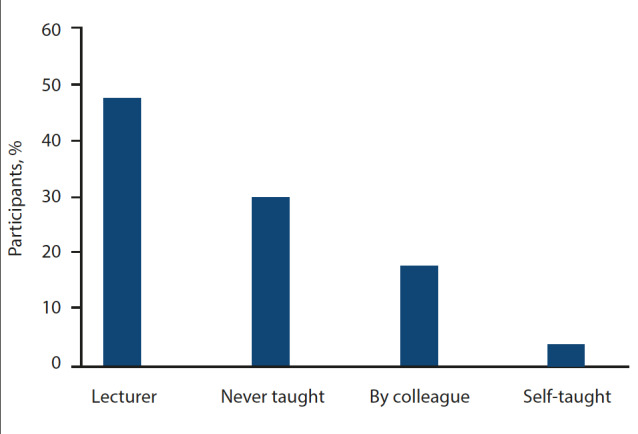
Participants’ metered-dose inhaler technique educator.

A third of study participants were never taught MDI technique. Of the remainder, nearly 50% were taught by lecturers at teaching institutions, whilst less than 20% were taught by colleagues (Fig. 2). There were no identifiable educational programmes in place dedicated to teaching HCPs on MDI technique and treatment protocols for patients on MDIs. Similarly, there was a lack of placebo MDIs in medical wards and emergency and pulmonology outpatient departments.

### HCP’s compliance in checking patients’ MDI technique, as well as demonstrating MDI technique to patients

**Fig. 3:**
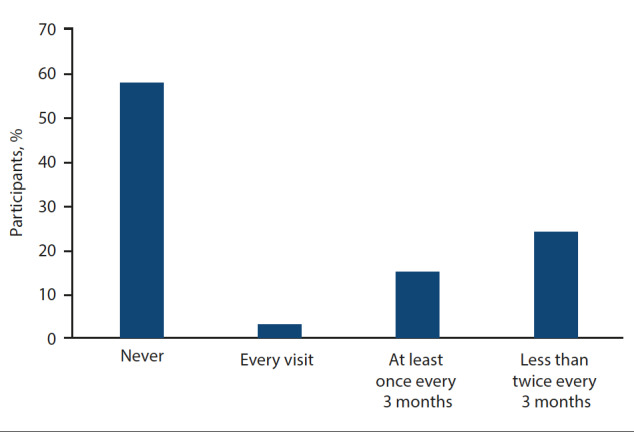
Healthcare professionals’ observation of patients’ metered-dose inhaler technique.

**Fig. 4:**
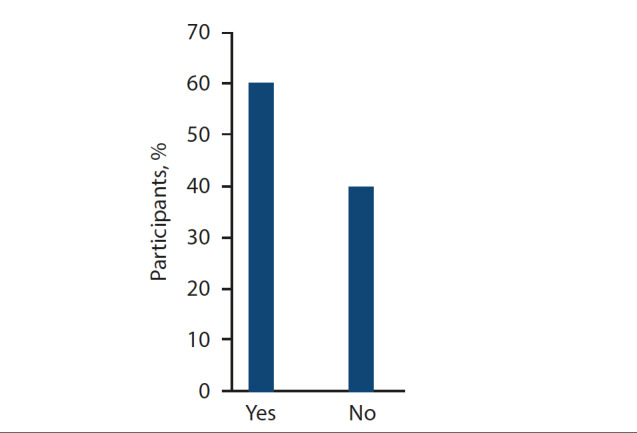
Demonstration of metered-dose inhaler technique to patients by healthcare professionals.

Fifty-seven percent of study participants disclosed that they did not observe patients’ MDI technique at every hospital-related visit (Fig. 3). Forty percent of study participants admitted to never demonstrating MDI technique to patients (Fig. 4). Although 50% of study participants stated that they routinely demonstrated inhaler technique to patients, the present study demonstrated poor MDI technique knowledge by HCPs; this translates to incorrect MDI technique being taught to patients.

In the present study, 45% of all participants felt that the responsibility to teach MDI technique was that of the treating doctor alone, whilst 21% were of the opinion that this was the nurses’ responsibility. No particular explanations for this stance were offered by study participants. The remaining participants expressed that all HCPs (nurse, treating doctor and pharmacist) involved in the care of the patient on inhaled therapy should educate patients regarding correct inhaler technique. There was no statistically significant difference between those who knew and those who did not know the correct MDI technique with respect to whether they demonstrated (p=0.0728) and/or observed their patients’ inhaler technique (p=0.1564) (Table 2).

## Discussion

The immense burden of infectious disease in sub-Saharan Africa has influenced governments to focus their limited resources on aggressive health campaigns in this area, resulting in the neglect of non-communicable diseases such as asthma and COPD. The increasing burden of COPD and asthma in our country is of great concern. Optimal management largely depends on the correct use of inhaler devices by patients, as well as repeated demonstrations and observation of patients’ inhaler technique by HCPs.

Inadequate knowledge of MDI use by HCPs has been documented globally, with similar findings in our local study. Plaza^[8]^ showed that only 14.2% of 1 514 physicians studied had adequate MDI technique. A South American-based study of 239 physicians showed that only 30% of all participants could demonstrate MDI technique correctly.^[9]^ The same study also demonstrated that 49% of the individuals interviewed in the pulmonology-cardiac specialist centre, and 19% of the individuals interviewed at a general internal medicine centre, were able to perform inhaler technique correctly.^[9]^ They postulated that the large difference between participants in the specialist centre and the general medical centre was due to the increased exposure of patients on inhaler therapy to those working at the specialist centre.^[9]^ Our study, with participants predominantly based in the internal medicine wards and emergency and outpatient departments, showed that only 16% of participants had adequate MDI technique. As only a small percentage of study participants were from the Division of Pulmonology (1.5%), we could not analyse whether there was a statistically significant difference between participants in the specialist unit v. those in general medicine. However, it is clear that overall there is poor knowledge of MDI use among HCPs. Contributing to this lack was the absence of placebo MDIs for demonstration of MDI technique in medical wards and emergency and pulmonology outpatient departments.

A third of our study participants were never taught MDI technique, with no identifiable educational programmes in place for this purpose. Rebuck *et al*.^[10]^ were of the opinion that postgraduate teaching programmes on MDI use should be determined by the needs of dayto-day patient care by HCPs. They conducted a study to determine if structured educational intervention v. none would be sufficient to teach postgraduate physicians inhaler technique skills that can be sustained over a long period. The 8-month follow-up of participants in the intervention group showed great improvement in the knowledge of MDI use compared with their baseline results (59% v. 42%; p<0.05). The intervention group performed better than the control group overall (59% v. 39%; p<0.05).^[10]^ Twenty percent of participants in our study were taught by fellow colleagues in an informal v. a structured teaching environment. The enforcement of structured teaching in the workplace as well as in our teaching institutions will improve knowledge of MDI use. The findings of our study should alert all HCPs, teaching institutions, senior personnel and managers in our hospitals to prioritise teaching MDI technique so that they may educate their patients and make a positive impact on disease control. Prioritising teaching MDI technique to HCPs will also sensitise them to the importance of compliance with treatment guidelines.

Plaza found that only 27.7% of 1 514 physicians checked their patients’ inhaler technique when inhaled therapy was prescribed.^[8]^ A study done in Zurich by Steurer-Stey *et al*. ^[11]^ found that 60% of physicians interviewed checked inhaler technique, and a significant two-thirds of the study participants said that patient education should be done at a pulmonology specialist centre. A survey of patients in a pulmonology unit in Japan found that only 17.1% of patients were given repeated physical demonstrations of MDI technique by a respiratory physician.^[12]^ Our study found that 57% of study participants admitted to never observing patients’ MDI technique, and 40% admitted to never demonstrating MDI technique to patients. It is important to note that although 50% of study participants stated that they routinely demonstrated inhaler technique to patients, only 16% of all study participants demonstrated correct inhaler technique. Therefore, incorrect inhaler technique is being taught to patients, further perpetuating the high morbidity and mortality associated with the underlying asthma or COPD.

It is important to note that although 50% of study participants stated that they routinely demonstrated inhaler technique to patients, only 16% of all study participants demonstrated correct inhaler technique. Therefore, incorrect inhaler technique is being taught to patients, further perpetuating the high morbidity and mortality associated with the underlying asthma or COPD.

Our study also found that having adequate knowledge of MDI technique did not translate to the participants complying with checking and demonstrating MDI use in real-world practice. Also of concern is that this lapse occurred despite the majority of study participants’ view that the treating doctor should be the sole responsible person for educating patients on MDI use. Our study population was much smaller than the above quoted studies, but concurs with their findings. All HCPs involved in the care of patients on inhaled therapy should ensure that they are familiar with correct inhaler technique and undertake the responsibility to educate their patients on MDI use routinely. This obligation is all the more important as we live in a developing country and therefore have less access to more userfriendly (and invariably more costly) devices. Our patients also have lower education levels and therefore require greater efforts to ensure that correct inhaler technique is learnt and sustained.

## Conclusion

Knowledge of correct MDI technique amongst HCPs and final-year medical students was poor. The study also revealed poor compliance amongst HCPs in observing patients’ inhaler technique and demonstrating correct inhaler technique to patients. We recommend that all teaching institutions, departments of internal medicine and emergency medicine and affected outpatient departments should have educational programmes in place dedicated to teaching correct MDI technique to HCPs and patients. We also recommend that HCPs should be familiar with treatment protocols for patients on inhaled therapy. Placebo inhaler devices should be easily accessible at all hospitals to enable HCPs to demonstrate MDI technique to patients.

## Figures and Tables

**Table 1:** Demographics of study participants (N=195)

Demographics	*n* (%)
Gender	
Female	130 (67)
Male	65 (33)
HCP (N=133)	
Medical doctor	96 (72)
Nurse	37 (28)
HCP years of experience* (N=133)	
>10	15 (11.3)
5 - 10	51 (38.3)
<5	67 (50.4)
Final-year medical students	62

HCP = healthcare professional

* Number of years after completion of undergraduate qualification.

**TABLE 2:** HCPs’ MDI technique and their compliance in checking and demonstrating MDI technique to patients on inhaled therapy

	Adequate MDI	Inadequate MDI
HCP characteristics	technique, *n*(%)	technique, *n*(%)
Total number of HCPs	32	101
Male	15 (47)	35 (35)
Female	17 (53)	66 (65)
Demonstrates MDI technique to patients		
Yes	8 (25)	72 (71)
No	24 (75)	29 (29)
Checks patients’ MDI technique		
Yes	4 (12.5)	53 (52)
No	28 (87.5)	48 (48)

HCP = healthcare professional

MDI = metered-dose inhaler
